# Church attendance, allostatic load and mortality in middle aged adults

**DOI:** 10.1371/journal.pone.0177618

**Published:** 2017-05-16

**Authors:** Marino A. Bruce, David Martins, Kenrik Duru, Bettina M. Beech, Mario Sims, Nina Harawa, Roberto Vargas, Dulcie Kermah, Susanne B. Nicholas, Arleen Brown, Keith C. Norris

**Affiliations:** 1 Center for Research on Men’s Health, Vanderbilt University, Nashville, Tennessee, United States of America; 2 Department of Internal Medicine, Charles R. Drew University School of Medicine and Science, Los Angeles, California, United States of America; 3 Department of Medicine, David Geffen School of Medicine at UCLA, Los Angeles, California, United States of America; 4 Department of Population Health Science, John D. Bower School of Population Health, University of Mississippi Medical Center, Jackson, Mississippi, United States of America; 5 Department of Internal Medicine, University of Mississippi Medical Center, Jackson, Mississippi, United States of America; 6 Department of Psychiatry, Charles R. Drew University School of Medicine and Science, Los Angeles, California, United States of America; 7 Department of Epidemiology, UCLA Fielding School of Public Health, Los Angeles, California, United States of America; 8 Department of Research and Life Science Institute, Charles R. Drew University School of Medicine and Science, Los Angeles, California, United States of America; Yokohama City University, JAPAN

## Abstract

**Importance:**

Religiosity has been associated with positive health outcomes. Hypothesized pathways for this association include religious practices, such as church attendance, that result in reduced stress.

**Objective:**

The objective of this study was to examine the relationship between religiosity (church attendance), allostatic load (AL) (a physiologic measure of stress) and all-cause mortality in middle-aged adults.

**Design, setting and participants:**

Data for this study are from NHANES III (1988–1994). The analytic sample (n = 5449) was restricted to adult participants, who were between 40–65 years of age at the time of interview, had values for at least 9 out of 10 clinical/biologic markers used to derive AL, and had complete information on church attendance.

**Main outcomes and measures:**

The primary outcomes were AL and mortality. AL was derived from values for metabolic, cardiovascular, and nutritional/inflammatory clinical/biologic markers. Mortality was derived from a probabilistic algorithm matching the NHANES III Linked Mortality File to the National Death Index through December 31, 2006, providing up to 18 years follow-up. The primary predictor variable was baseline report of church attendance over the past 12 months. Cox proportional hazard logistic regression models contained key covariates including socioeconomic status, self-rated health, co-morbid medical conditions, social support, healthy eating, physical activity, and alcohol intake.

**Results:**

Churchgoers (at least once a year) comprised 64.0% of the study cohort (*n* = 3782). Non-churchgoers had significantly higher overall mean AL scores and higher prevalence of high-risk values for 3 of the 10 markers of AL than did churchgoers. In bivariate analyses non-churchgoers, compared to churchgoers, had higher odds of an AL score 2–3 (OR 1.24; 95% CI 1.01, 1.50) or ≥4 (OR 1.38; 95% CI 1.11, 1.71) compared to AL score of 0–1. More frequent churchgoers (more than once a week) had a 55% reduction of all-cause mortality risk compared with non-churchgoers. (HR 0.45, CI 0.24–0.85) in the fully adjusted model that included AL.

**Conclusions and relevance:**

We found a significant association between church attendance and mortality among middle-aged adults after full adjustments. AL, a measure of stress, only partially explained differences in mortality between church and non-church attendees. These findings suggest a potential independent effect of church attendance on mortality.

## Introduction

While interest in the relationship between religion and health is almost as old as humanity, the science relating these rich concepts has grown considerably in the past two decades as increasing numbers of peer-reviewed articles have been reported with results from studies exploring links between religion and various dimensions of physical or mental health [1–3]. Interestingly, the results reported in this body of literature have been mixed. A number of studies have indicated that religion can be beneficial to health [3–8]. However, it is also noteworthy that other studies have found religion to have no effect or, in some cases, a negative association with health outcomes [1, 2]. These apparently conflicting findings can be attributed to multiple sources, including differences in the operationalization and measurement of these multidimensional factors. It has been suggested that the beneficial effects of religiosity on the attitudes, motivations, goals, social interactions, and perceptions of individuals about wellness can be assessed by church attendance [9, 10]. Prior analyses of the National Health and Nutrition Examination Survey (NHANES) III linked mortality dataset [1988–1994] have suggested that the association between church attendance and longevity among adult participants may be mediated by other risk factors including health behaviors and inflammation [11].

Thus, the effect of religiosity or church attendance on health may be mediated in part by a healthy lifestyle, social cohesion or other factors such as mitigating stress. A related potential mediator in this context is allostatic load (AL), a term that refers to the accumulation of physiological perturbations as a result of repeated or chronic stressors in daily life [12–14]. The measurement of AL may include levels of hormones secreted in response to stress and/or related clinical/biologic markers that indirectly reflect the effects of such hormones on the body [12]. Higher AL has been associated with premature morbidity and mortality in a range of studies and populations [15], and growing distress was recently reported to underlie the significant rise in the death rate of white middle-aged Americans [16]. Because religiosity may have a positive impact on stressors, AL levels could vary by level of religiosity.

We posited that religiosity would be associated with lower mortality risk, in part, through AL. Thus, using longitudinal follow-up NHANES III baseline data, we investigated whether religiosity in adults as estimated by church attendance was associated with AL and/or mortality and whether associations with mortality were mediated by AL. We hypothesized that church attendance would be inversely related to AL levels, after adjusting for socioeconomic measures, health insurance status and health behaviors. Given the recent findings of distinct changes in mortality over the last 15 years for middle-aged adults [16], we focused our primary analysis on persons 40–65 years of age.

## Material and methods

### Survey design and data collection

Data for our study were drawn from NHANES III (1988–1994), which included approximately 40,000 persons from 89 random US locations [17]. The NHANES III uses a stratified, multistage probability design to create a nationally representative sample of the civilian, non-institutionalized persons [17]. The NHANES III also conducts household interviews and collects sociodemographic and clinical information including height, weight and blood pressure, as well as blood samples for specific biochemical analyses [18]. Because NHANES III data were publicly available and subjects were de-identified, the project was exempt from IRB review.

**Fig 1:**
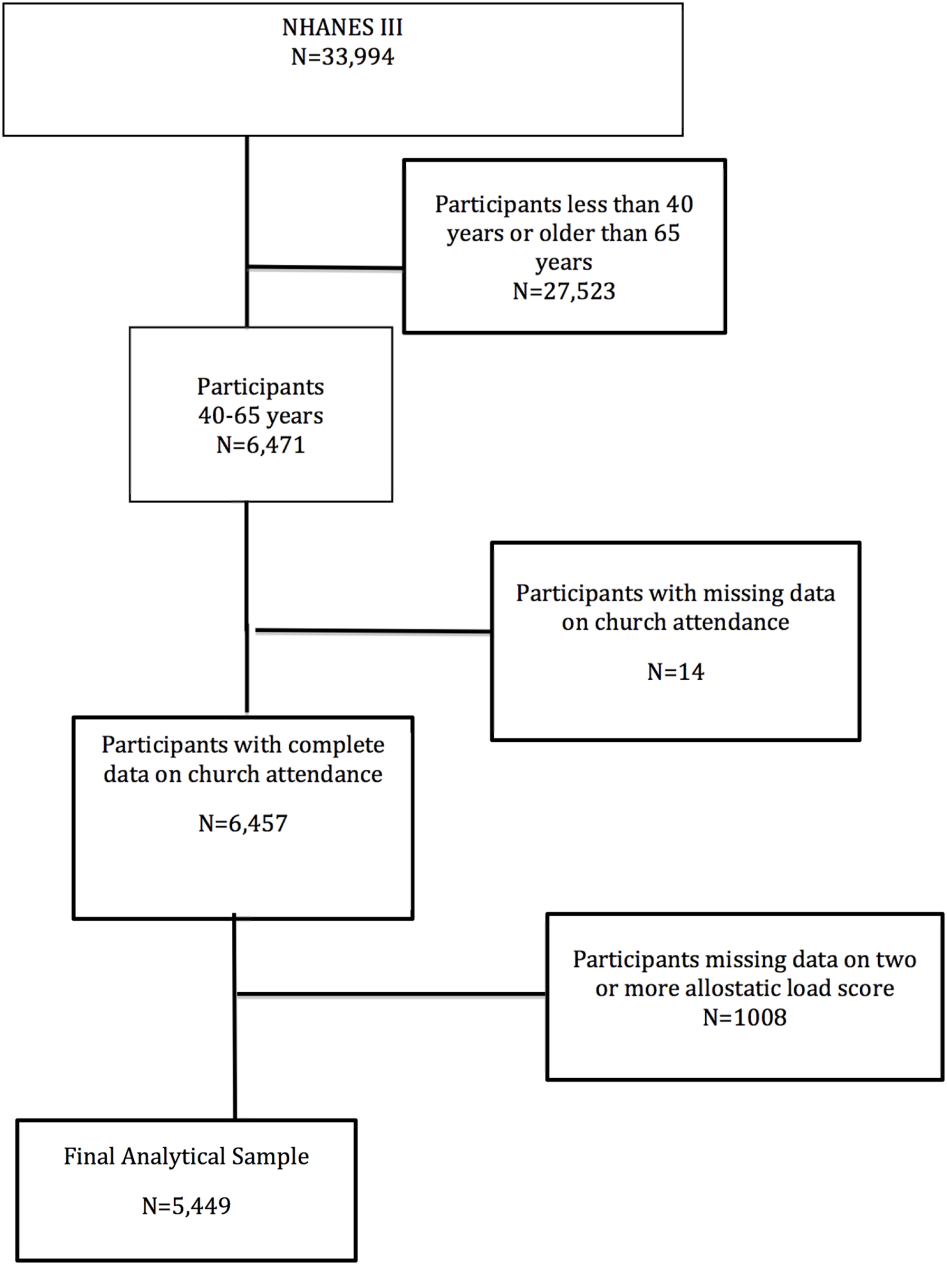
Algorithm used to define the study cohort.

Our study population was derived from NHANES III adult participants aged 40–65 years at the time of interview (n = 6,471). Mean duration of follow up for survival was 14.2 years [11]. Participants who were missing data for two or more components of the AL score (n = 1008), were excluded. Participants with missing data on church attendance were also excluded (n = 14). The final study cohort was comprised of 5,449 participants (Fig 1). A sensitivity analysis was also conducted with all adults aged ≥40 years (final analytic sample = 8,835; supplemental files) and for frequency of church attendance.

### Study variables

Allostatic load was calculated as a summative measure derived from values for 10 clinical/biologic markers available in NHANES III that have previously been reported to represent physiological dysregulation [19, 20]. These included: cardiovascular (systolic blood pressure, diastolic blood pressure, total cholesterol/high density lipoprotein (HDL) ratio, homocysteine); nutritional/inflammatory markers (albumin, C-reactive protein); and metabolic (waist-hip ratio, glycated hemoglobin) [19, 20]. We then created and compared three categories of AL based on the number of clinical/biologic markers present (low 0–1, medium 2–3, and high > = 4).

The NHANES III Linked Mortality File was used to estimate race-specific, non-injury-related death rates for NHANES III participants using a probabilistic matching algorithm, linked to the National Death Index through December 31, 2006 [21] and provided up to 18 years follow-up (mean [SE] 14 [0.2] years).

The church attendance measure was derived from an interview question asking respondents “How often do you attend church or religious services? (per year)”. Responses ranged from not at all to 1095 times a year. Select covariates were included in both sets of models. Behavioral covariates were included physical activity (any vs. none), smoking status (current, former, never), alcohol use (non-drinker, 1–30 drinks/month, >30 drinks per month), and the Healthy Eating Index (HEI) which was scored from 0–100 (higher score represents healthier eating) [19]. Socioeconomic status (SES) covariates included three variables. Education was categorized as three- variables denoting whether an individual completed <9, 9–12, and >12 years of education. Health insurance was coded as a dichotomous variable indicating whether or not a participant was insured. Income was measured by the poverty-income ratio (PIR), an income-to-needs variable measuring the ratio of household income to the US poverty threshold based on each respondent’s family size and composition at the time of the NHANES III examination [19]. Self-rated health was assessed with a single item, “How would you rate your overall health?” with categories ranging from excellent to poor [22, 23]. In this study, self-rated health was categorized into three groups: “Excellent or Very Good”, “Good”, and “Fair or Poor” [24]. We also examined social support indicators [25] as potential covariates using the following questions: a) “In a typical week, how many times do you talk on the telephone with family, friends, or neighbors?”, b) “How often do you get together with friends or relatives; I mean things like going out together or visiting in each other's homes? (per year)”, c) “About how often do you visit with any of your other neighbors, either in their homes or in your own? (per year)”, The natural logarithmic transformation of each social support variable was used in our analyses to compensate for skewness.

### Statistical analyses

Study population characteristics were described by church attendance, using means and standard errors for continuous variables and proportions for categorical variables. All estimates were weighted to adjust for the differential probabilities of sampling and non-response, to represent the total civilian, non-institutionalized US population. Therefore, actual sample sizes are not reported along with the percentages. Estimates derived from a sample size smaller than the recommended lower limit in the NHANES analytic guidelines were considered unreliable [26]. Logistic regression was used to estimate bivariate AL models and four sequential Cox proportional hazard regressions were constructed to examine the association between the level of church attendance and all-cause mortality. Model 1 included adjustment for race, age, sex, and chronic conditions (asthma, chronic obstructive pulmonary disease, non-skin cancer, thyroid disease, rheumatoid arthritis), Model 2 added SES (education, poverty-income ratio, and health insurance status), Model 3 added health behaviors (smoking status, alcohol use, physical activity, and HEI) to Model 2, Model 4 added AL score to Model 3, and Model 5 added self-rated health and social support indicators to Model 4. Results are expressed as hazard ratios (HRs) with 95% confidence intervals (CIs). All analyses were performed using SAS v 9.4 (Research Triangle Park, NC), and all estimates and statistical tests were adjusted for the complex NHANES survey design.

## Results

### Baseline characteristics

Churchgoers (at least once a year) comprised 64.0% of the study cohort (*n* = 3,782) and non-churchgoers (no church at all) comprised 36.0% (*n* = 1,667). The mean (SE) age of the overall cohort was 51±0.2 years and did not differ between churchgoers and non-churchgoers (Tables 1 and 2). Baseline characteristics revealed churchgoers had a better socioeconomic and health behavior profile. Specifically, they were more likely to have higher levels of educational attainment, lower levels of poverty, increased physical activity, reduced rates of smoking and drinking, and a healthier eating index. More than 50% of the churchgoers reported they had “Excellent/Very Good Health”. Overall, non-churchgoers spent more time seeking social support from family, friends, relatives, and neighbors than churchgoers. (Table 1).

**Table 1:** Baseline characteristics of NHANES III participants by self-reported church attendance.

	Total (n = 5449)	Churchgoers (At least once a year) (n = 3782)	Non-churchgoers (n = 1667)	*P value*
Demographics				
Mean age (SE) years	**51(0.2)**	**51(0.3)**	**51(0.3)**	***0*.*405***
Race/Ethnicity				***<0*.*001***
White	**2280**	**1388(75.7)**	**892(84.6)**	
Black	**1466**	**1146(11.6)**	**320(6.0)**	
Hispanic	**1458**	**1091(4.9)**	**367(2.9)**	
Other	**245**	**157(7.7)**	**88(6.4)**	
Sex				***<0*.*0001***
Male	**2612**	**1646(44.9)**	**966(54.7)**	
Female	**2837**	**2136(55.2)**	**701(45.3)**	
Education				***0*.*0004***
<9 years (%)	**1338**	**935(11.1)**	**403(13.1)**	
9–12 years (%)	**2246**	**1524(41.0)**	**722(48.4)**	
>12 years (%)	**1577**	**1140(47.9)**	**437(38.4)**	
Poor (poverty-income ratio<2) (%)	**2129**	**1484(22.8)**	**645(28.1)**	***0*.*013***
No health insurance (%)	**816**	**545(8.2)**	**271(13.1)**	***0*.*003***
Self-rated health				***0*.*018***
Excellent/Very Good (%)	**2008**	**1387(51.6)**	**621(46.4)**	
Good (%)	**1970**	**1381(33.1)**	**589(33.9)**	
Fair/Poor (%)	**1469**	**1013(15.2)**	**456(19.7)**	
Social Support Mean (SE)				
In a typical week, how many times do you talk on the telephone with family, friends, or neighbors? (per week)	**5429**	**93.4(15.6)**	**208.9(45.1)**	***0*.*09***
How often do you get together with friends or relatives; I mean things like going out together or visiting in each other's homes? (per year)	**5449**	**100.8(3.1)**	**104.0(8.1)**	***0*.*03***
About how often do you visit with any of your other neighbors, either in their homes or in your own? (per year)	**5443**	**55.5(4.1)**	**59.7(8.1)**	***0*.*01***
Comorbidities (non-CV related)				
Lung disease (%)	**447**	**269(7.6)**	**178(10.9)**	***0*.*006***
Cancer (%)	**185**	**130(4.1)**	**55(4.0)**	***0*.*901***
Thyroid disease (%)	**342**	**250(7.0)**	**92(6.7)**	***0*.*797***
Rheumatoid arthritis (%)	**290**	**200(4.4)**	**90(4.9)**	***0*.*535***
Systemic lupus erythematosus (%)^a^	**19**	**13(0.3)**	**6(0.7)**	***0*.*30***
Asthma (%)	**414**	**270(8.1)**	**144(9.0)**	***0*.*501***
Health Behaviors				
Tobacco Use				***<0*.*0001***
Current smokers (%)	**1545**	**910(22.1)**	**635(35.3)**	
Former smokers (%)	**1616**	**1093(32.4)**	**509(29.5)**	
Never smokers (%)	**2288**	**1779(45.5)**	**509(29.5)**	
Physically active (%)	**3725**	**2633(79.1)**	**1092(74.9)**	***0*.*011***
Alcohol Use				***0*.*001***
Non-drinkers (%)	**2824**	**2072(47.5)**	**752(43.9)**	
1–30 alcoholic drinks/month (%)	**2249**	**1521(46.7)**	**728(44.2)**	
>30 alcoholic drinks/month (%)	**375**	**189(5.8)**	**186(11.9)**	
Mean (SE) Healthy Eating Index score	**5287**	**65(0.4)**	**63(0.6)**	***0*.*007***

^a^Estimate is unreliable, as the sample size was smaller than that recommended in the NHANES analytic guidelines for the design effect and estimated proportion.[22, 23]

The data presented are the weighted percentages, so they may not add up to 100. SE: standard error; CV-cardiovascular

**Table 2:** Baseline allostatic load components of NHANES III participants.

	Total (n = 5449)	Churchgoers (*n* = 3782)	Non-churchgoers (*n* = 1667)	*P* value
***Allostatic Load Components*** (% of each subgroup with “high risk” values)^a^				
Systolic blood pressure	1179	811(14.3)	368(18.0)	0.017
Diastolic blood pressure	540	375(7.8)	165(9.7)	0.063
Waist/hip ratio	4284	2950(74.3)	1334(77.5)	0.143
HDL	1365	917(24.2)	448(28.0)	0.022
Total cholesterol/HDL ratio	1906	1291(33.2)	615(37.9)	0.030
Glycated hemoglobin	1656	1191(20.2)	465(20.6)	0.750
Heart Rate	206	130(3.0)	76(4.2)	0.144
Albumin	710	521(11.6)	189(9.0)	0.086
C-reactive protein	2091	1458(32.1)	633(32.7)	0.769
Body Mass Index	1702	1209(26.3)	493(28.4)	0.295
Mean (SE) allostatic load score [range 0–10]	5449	2.5(0.1)	2.6(0.1)	0.008

^a^High-risk values were defined as Systolic blood pressure>140 mmHg; Diastolic blood pressure>90 mmHg; waist/hip ratio>0.9(Males) waist/hip ratio>0.85(Females); HDL<40; Chol/HDL>5; HbA1c>5.7; Heart rate>90; albumin <3.8; C-reactive protein≥0.3; Body mass index >30; SE: standard error.

### Allostatic load

In unadjusted analyses, participants who were non-churchgoers had significantly higher rates of high-risk values for 3 of the 10 markers of AL (systolic blood pressure, HDL cholesterol and total cholesterol/ HDL ratio) and higher mean AL scores (p = 0.008) than did those who reported at least monthly church attendance. There was no significant difference in AL based on frequency of church attendance (both at least weekly and at least monthly differed from no church attendance but not from each other, data not shown). Since we did not find a statistically significant dose-response relationship with frequency of church attendance, we compared those who attended some church to non-churchgoers (Table 3). The regression analysis presented in Table 3 revealed that compared to churchgoers, non-churchgoers had a higher AL, with odds ratio (OR) and 95% confidence interval (CI) of 1.24 (1.01–1.50) for 2–3 AL components vs. 0–1 and 1.38 (1.11–1.71) for ≥4 components vs. 0–1.

**Table 3:** Elevated allostatic load by church attendance.

Allostatic load Score^a^	Odds ratio (95% CI) of higher allostatic load score of No Church vs. Some Church
**2–3** **vs. 0–1**	1.24 (1.01–1.50) ^b^
**≥ 4 vs. 0–1**	1.38 (1.11–1.71) ^c^

^a^Total allostatic load score was categorized using 2 different cutoffs (0–1, 2–3, and ≥4). Sum of the components include diastolic BP, systolic BP, HbA1c, waist to hip ratio, HDL, total cholesterol/HDL ratio, albumin, C-reactive protein, heart rate and BMI concentrations.

^b^*P* = 0.04

^c^*P* = 0.004

### Mortality

**Fig 2:**
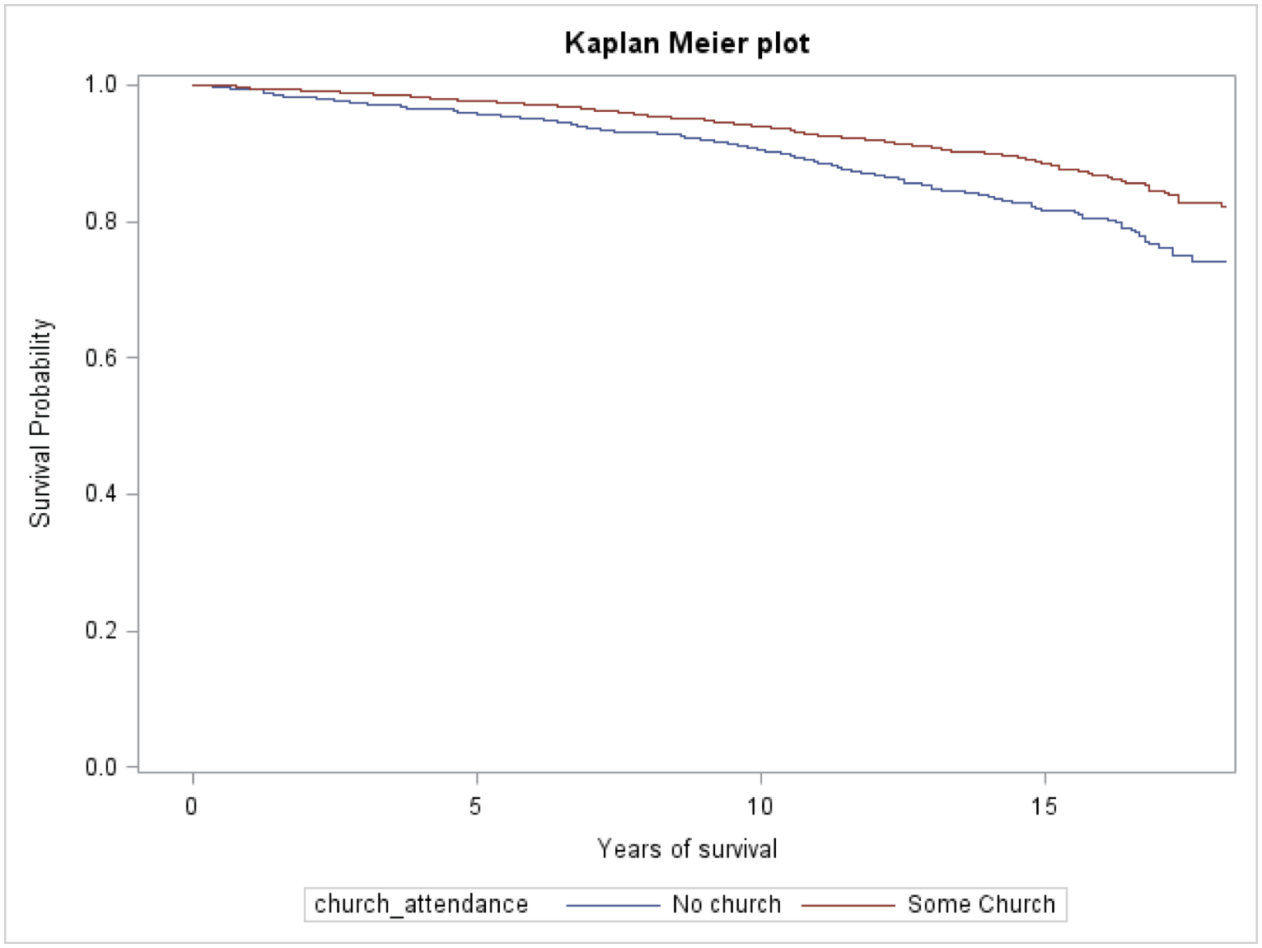
Kaplan-Meier curves for all-cause mortality by church attendance.

The hazard models for all-cause mortality are presented in Table 4. Compared to non-churchgoers, churchgoers who attended church more than weekly had a 49% unadjusted reduced risk for all-cause mortality. After adjustment for age, sex, race, and chronic medical conditions churchgoers had a 46% reduction in all-cause mortality. Sequential adjustments for SES (education, health insurance, and PIR; hazard ratio (HR) 0.54), health behaviors (smoking status, physical activity, alcohol use and healthy eating index; (HR) 0.55) minimally affected the HR. Adjustment for stress/cardiovascular indicators (AL) attenuated the HR to 0.60 (0.37–0.95). After controlling for social support indicators and self-rated health to create the fully adjusted model, the HR for churchgoers who attended church more than weekly was 0.45 (0.24–0.85). (Fig 2). For the survival models, we tested and found no significant interaction between church attendance and race or sex, and no effect of AL, stratified by tertiles, on the relationship of church attendance with mortality (data not shown).

**Table 4:** Hazard ratio for all-cause mortality by church attendance.

	Unadjusted	Adjusted				
		Model 1	Model 2	Model 3	Model 4	Model 5
**No church at all** **(N = 1667)**	Reference	Reference	Reference	Reference	Reference	Reference
**Less than Weekly** **(N = 1475)**	0.62 (0.49–0.77)	0.66 (0.53–0.83)	0.65 (0.50–0.83)	0.68 (0.53–0.86)	0.69 (0.53–0.89)	0.59 (0.41–0.85)
**Weekly** **(N = 1720)**	0.64 (0.52–0.79)	0.65 (0.51–0.82)	0.64 (0.48–0.85)	0.73 (0.54–0.98)	0.75 (0.56–1.00)	0.61 (0.39–0.95)
**More than weekly** **(N = 587)**	0.51 (0.38–0.69)	0.54 (0.38–0.76)	0.54 (0.37–0.78)	0.55 (0.35–0.86)	0.60 (0.37–0.95)	0.45 (0.24–0.85)

Model 1 adjusts for age, sex, and race.

Model 2 adds education, poverty-income ratio, and health insurance status to the covariates in Model 1.

Model 3 adds health behaviors and the healthy eating index score to the covariates in Model 2.

Model 4 adds allostatic load score to the covariates in Model 3.

Model 5 adds social support and self-rated health to the covariates in Model 4.

(All adjusted models adjusted for asthma, chronic obstructive pulmonary disease, non-skin cancer, thyroid disease, rheumatoid arthritis)

### Sensitivity analysis

An NHANES sensitivity analysis using the same strategies for participant ascertainment but without restriction by older age (all participants ≥40 years) revealed similar findings to the main analyses (S1–S4 Tables). Odds ratios (OR; 95% CI) for AL scores of 2–3 and ≥ 4 compared to 0–1, by church attendance were OR 1.14 (0.94–1.38), and OR 1.35 (1.11–1.63) respectively. Similar to the primary analysis, a fully adjusted Cox proportional hazard regression model demonstrated a 34% decreased risk 0.66 (0.49–0.87) for all-cause mortality for churchgoers who attended church more than weekly compared to non-churchgoers. Again, statistically significant differences were not noted when we assessed the impact of the frequency of church attendance.

## Discussion

This study examined the association of church attendance with prevalent AL and all-cause mortality in a large multiethnic sample. We found that the lack of church attendance (a surrogate for religiosity) was associated with a greater likelihood of having an elevated AL (a measure of physiologic dysregulation) and all-cause mortality that persisted after further adjustment of socio-demographic, clinical and laboratory confounders. The higher adjusted mortality rate for non-churchgoers was slightly attenuated after controlling for AL, but remained statistically significant, suggesting the potential influence of religiosity independent of mediation by AL.

Our findings are consistent with prior studies that linked AL with mortality [19, 27]. They also build upon, but are distinct from, the work of Gillum et al. who found a lower unadjusted mortality rate among weekly and more-than-weekly church attendees compared with non-attendees using NHANES III participants ages 40 and over; but this association was no longer significant after controlling for common health-related mediating factors [11]. In contrast to the analysis by Gillum and colleagues, our primary analyses focus on premature mortality among participants aged 40–65, who represent a particularly vulnerable group, and we examined AL as a potential mediator of the relationship between church attendance and mortality. We also performed a sensitivity analysis of mortality in persons ≥ 40 years of age to examine whether the longer follow up in our analyses–14.2 years in contrast to 8.5 years in the Gillum paper—resulted in a different association between church attendance and mortality. Our findings are consistent with a possible independent effect of church attendance on mortality that is consistent with data recently reported by Li et al. [28] in over 74,000 women in the Nurses’ Health Study. They also indicate that church attendance and possibly religiosity may mitigate the effect of stressors on physiologic dysregulation.

Our findings support the overall hypothesis that increased religiosity (as determined by church attendance) is associated with less stress (as assessed by AL) and enhanced longevity. The positive relationship of church attendance with both reduced AL and longevity suggest religiosity can affect two well-described objective health parameters. The role of church attendance in enhancing longevity may be mediated through several causal pathways linking religion and physical health such as psychological traits, lifestyle decisions, social conditions and social networks as described by Koenig [29], Li et al. [28] and Hummer et al. [22]. Thus, we also controlled for self reported health which has been reported to be a powerful indicator of mortality [23] and time spent seeking social support from family, friends, relatives, and neighbors [22] in an effort to address additional key elements not incorporated by allostatic load, our physiologic measure of chronic stress. Even with the adjustments the association of church attendance with reduced mortality remained constant.

Health benefits of religiosity may also be attributed, in part, to its impact on two less commonly cited domains, compassion [30] and a sense of holiness [31]. Compassion has a long history of association with religiosity [32] and has been reported to mediate some of the psychosocial health benefits of religiosity and social relationships through its related generosity and altruism [33], as well as reducing stress and/or enhancing coping skills [34]. Holiness gives meaning and purpose to life [35] and inspires commitment to something greater than self [36]. Holiness instills love, joy, peace, hope and fulfillment [37], fosters a sense of inter-connectedness with others [38], promotes a sense of wholeness in life [39], and engenders a greater personal relationship with a higher power [40], possibly explaining the health benefits attributed to religiosity [41, 42].

Although these analyses support the literature linking religiosity and mortality, we found no evidence that the actual frequency of church attendance offered similar protection as no differences in AL or mortality were found between those attending church at least weekly and those attending church at least monthly. Allostatic load has been described as a multifactorial concept that links an individual with their local environmental stressors and the balance of different components may eventually manifest as a distinct disease(s). Specifically, individual gene polymorphisms juxtaposed with life experiences, environmental stressors and either adaptive behavioral responses (e.g. exercise, good nutrition) or maladaptive behavioral responses (e.g. substance abuse) influence the development and progression of many disease states [12]. An increased AL and associated organ dysfunction may result from increased frequency or degree of select environmental stressors, ability or inability to adapt to such stressors over time, and the psychological impact of stress such as anticipation (e.g., worrying about a possible stressful event in the future that may or may not take place) and/or memories of prior stressors (e.g., post-traumatic stress disorder) [12]. Similarly, Cohen and colleagues reported that environment stress can result in maladaptive coping mechanisms such as negative emotional states, superimposed psychological distress and/or the adoption of unhealthy health beliefs and behaviors [43]. The confluence of these psychological and behavioral responses may lead to long-term physiological changes that are characterized by increased AL and ultimately organ dysfunction. Our findings that the compilation of stress responses assessed by AL is more common among non-church attendees than church attendees are supportive of these concepts and consistent with Powell et al. who suggested religiosity/spirituality is strongly linked to improved health outcomes in the general population [44]. Health outcomes may also differ across different religious groups and practices. For example, Seventh-day Adventists have been reported to experience lower risks of diabetes mellitus, hypertension, and arthritis. It has been suggested these patterns are likely due to the Adventists’ predominantly vegetarian diet; however, other more religious-based mechanisms cannot be excluded [45].

Our study had several limitations. The clinical/biologic markers assessed, while commonly studied, are a substantive but not a complete measure of physiological dysregulation, and are unlikely to fully capture alterations in immune function, inflammatory responses, or neuroendocrine function, such as that regulated by the hypothalamic-pituitary-adrenal (HPA) axis. The NHANES III dataset does not include lab values on markers of the HPA axis such as cortisol and epinephrine. It is likely that due to lack of data on duration and/or severity of components of the overall AL measure our crude summary score may not be optimal. While we adjusted for many biologic and environmental factors, we cannot exclude residual confounding and since this is an observational study, one cannot infer causality. It is also possible that the health benefits of religiosity have been underestimated because of the crude or imprecise measurement. Church attendance can represent a public expression of religiosity but does not account for private practices such as prayer. Lastly, while we have postulated that church attendance in this survey may reflect religiosity, and we attempted to adjust for the potential positive impact of church attendance on socialization or social biases against those not attending church [22], we cannot exclude residual confounding from factors such as disease severity/duration or other aspects of social support not included in our model. Each of these might affect the observed differences in the associations for both stress and mortality among church attendees compared to non-church attendees.

In conclusion, we found a significant relationship between church attendance and mortality in middle-aged (≥40–65 yrs) adults NHANES III participants with an extended mean follow up time of 14 years. This relationship remained significant even after adjustment for education, poverty status, health insurance status, self-rated health, social support, and AL, suggesting a potential independent effect of religiosity on mortality. Similar findings were also noted in a secondary analysis of NHANES III participants ≥ 40 years old. Our results underscore the potential importance of church attendance as a surrogate for religiosity as a mediator of health and lifespan. The increased attention to religiosity and other faith-related factors by health professionals and scientists is warranted by these findings and those from similar studies [46]. Results from this study contribute to the existing body of evidence and support the need for more rigorous prospective studies to explore causal relationships of religiosity and health.

## Supporting information

S1 Fig Supplemental Figure 1. (PDF)

S2 Fig Supplemental Figure 2. (PDF)

S1 Table Baseline characteristics of NHANES III population: Demographics. (DOCX)

S2 Table Baseline characteristics of NHANES III population: Allostatic load components. (DOCX)

S3 Table Elevated allostatic load by church attendance. (DOCX)

S4 Table Hazard ratio for all-cause mortality by church attendance. (DOCX)

## References

[1] Ferraro KF, Kim S. Health benefits of religion among Black and White older adults? Race, religiosity, and C-reactive protein. Social science & medicine. 2014;120C:92–9. 10.1016/j.socscimed.2014.08.030 25226450

[2] George LK, Kinghorn WA, Koenig HG, Gammon P, Blazer DG. Why gerontologists should care about empirical research on religion and health: transdisciplinary perspectives. The Gerontologist. 2013;53(6):898–906. 10.1093/geront/gnt002 23442382

[3] Koenig HG, McCullough MF, Larson DB. Handbook on Religion and Health. Oxford: Oxford University Press; 2001.

[4] Buck AC, Williams DR, Musick MA, Sternthal MJ. An examination of the relationship between multiple dimensions of religiosity, blood pressure, and hypertension. Social science & medicine. 2009;68(2):314–22. 19019516 10.1016/j.socscimed.2008.10.010 PMC2654362

[5] Gillum RF. Frequency of attendance at religious services and leisure-time physical activity in American women and men: the Third National Health and Nutrition Examination Survey. Annals of behavioral medicine: a publication of the Society of Behavioral Medicine. 2006;31(1):30–5. 16472036 10.1207/s15324796abm3101_6

[6] Gillum RF. Frequency of attendance at religious services, overweight, and obesity in American women and men: the Third National Health and Nutrition Examination Survey. Annals of epidemiology. 2006;16(9):655–60. 10.1016/j.annepidem.2005.11.002 16431132

[7] Gillum RF, Ingram DD. Frequency of attendance at religious services, hypertension, and blood pressure: the Third National Health and Nutrition Examination Survey. Psychosomatic medicine. 2006;68(3):382–5. 10.1097/01.psy.0000221253.90559.dd 16738068

[8] Seeman TE, Dubin LF, Seeman M. Religiosity/spirituality and health. A critical review of the evidence for biological pathways. The American psychologist. 2003;58(1):53–63. 12674818 10.1037/0003-066x.58.1.53

[9] Koenig H, King D, Carson VB. Handbook of religion and health: Oxford University Press; 2012.

[10] Singh DK, Ajinkya S. Spirituality and religion in modern medicine. Indian journal of psychological medicine. 2012;34(4):399–402. 10.4103/0253-7176.108234 23723556 PMC3662145

[11] Gillum RF, King DE, Obisesan TO, Koenig HG. Frequency of attendance at religious services and mortality in a US national cohort. Annals of epidemiology. 2008;18(2):124–9. 10.1016/j.annepidem.2007.10.015 18083539 PMC2659561

[12] McEwen BS. Protective and damaging effects of stress mediators. The New England journal of medicine. 1998;338(3):171–9. 10.1056/NEJM199801153380307 9428819

[13] Seeman TE, Singer BH, Rowe JW, Horwitz RI, McEwen BS. Price of adaptation—allostatic load and its health consequences. MacArthur studies of successful aging. Archives of internal medicine. 1997;157(19):2259–68. 9343003

[14] Stewart JA. The detrimental effects of allostasis: allostatic load as a measure of cumulative stress. Journal of physiological anthropology. 2006;25(1):133–45. 16617218 10.2114/jpa2.25.133

[15] Hatch SL. Conceptualizing and identifying cumulative adversity and protective resources: implications for understanding health inequalities. The journals of gerontology Series B, Psychological sciences and social sciences. 2005;60 Spec No 2:130–4. 10.1093/geronb/60.special_issue_2.s130 16251584

[16] Case A, Deaton A. Rising morbidity and mortality in midlife among white non-Hispanic Americans in the 21st century. Proc Natl Acad Sci U S A. 2015;112(49):15078–83. 10.1073/pnas.1518393112 26575631 PMC4679063

[17] Centers for Disease Control and Prevention. Plan and operation of the Third National Health and Nutrition Examination Survey, 1988–94: series 1: programs and collection procedures. Vital Health Statistics. 1994;1(32):1–407. 7975354

[18] National Center for Health Statistics, Centers for Disease Control and Prevention. Plan, and Operation of the Third National Health and Nutrition Examination Survey (NHANES III, 1988–94): Reference Manuals and Reports: Weighting and Estimation Methodology Report. Hyattsville, MD: US Dept of Health and Human Services, Public Health Service, Centers for Disease Control and Prevention; 1998.

[19] Duru OK, Harawa NT, Kermah D, Norris KC. Allostatic load burden and racial disparities in mortality. Journal of the National Medical Association. 2012;104(1–2):89–95. 22708252 10.1016/s0027-9684(15)30120-6 PMC3417124

[20] Geronimus AT, Hicken M, Keene D, Bound J. "Weathering" and age patterns of allostatic load scores among blacks and whites in the United States. Am J Public Health. 2006;96(5):826–33. 10.2105/AJPH.2004.060749 16380565 PMC1470581

[21] National Center for Health Statistics, Office of Analysis and Epidemiology. Third National Health and Nutrition Examination Survey (NHANES III) Linked Morality File, Mortality follow-up through 2006: Matching Methodolgy May 2009. Hyattsville, MD: US Dept of Health and Human Services, Public Health Service, Centers for Disease Control and Prevention; 2009 [http://www.cdc.gov/nchs/data/datalinkage/matching_methodology_nhanes3_final.pdf.

[22] Hummer RA, Rogers RG, Nam CB, Ellison CG. Religious involvement and U.S. adult mortality. Demography. 1999;36(2):273–85. 10332617

[23] Ferraro KF, Farmer MM. Utility of health data from social surveys: Is there a gold standard for measuring morbidity? American Sociological Review. 1999;64(2):303–15.

[24] Jones SA, Wen F, Herring AH, Evenson KR. Correlates of US adult physical activity and sedentary behavior patterns. J Sci Med Sport. 2016;19(12):1020–7. 10.1016/j.jsams.2016.03.009 27053434 PMC5036978

[25] Beydoun MA, Beydoun HA, Mode N, Dore GA, Canas JA, Eid SM, et al Racial disparities in adult all-cause and cause-specific mortality among us adults: mediating and moderating factors. BMC Public Health. 2016;16(1):1113 10.1186/s12889-016-3744-z 27770781 PMC5075398

[26] U. S. Department of Health and Human Services., National Center for Health Statistics. Analytic and Reporting Guidelines: The Third National Health and Nutrition Examination Survey, NHANES III (1988–94). Hyattsville, MD: Centers for Disease Control and Prevention; 1996.

[27] Karlamangla AS, Singer BH, Seeman TE. Reduction in allostatic load in older adults is associated with lower all-cause mortality risk: MacArthur studies of successful aging. Psychosomatic medicine. 2006;68(3):500–7. 10.1097/01.psy.0000221270.93985.82 16738085

[28] Li S, Stampfer MJ, Williams DR, VanderWeele TJ. Association of Religious Service Attendance With Mortality Among Women. JAMA Intern Med. 2016;176(6):777–85. 10.1001/jamainternmed.2016.1615 27183175 PMC5503841

[29] Koenig HG. Religion, spirituality, and health: the research and clinical implications. ISRN psychiatry. 2012;2012:278730 10.5402/2012/278730 23762764 PMC3671693

[30] Steffen PR, Masters KS. Does compassion mediate the intrinsic religion-health relationship? Annals of behavioral medicine: a publication of the Society of Behavioral Medicine. 2005;30(3):217–24. 16336073 10.1207/s15324796abm3003_6

[31] Martins D, Nicholas NA, Shaheen M, Jones L, Norris K. The development and evaluation of a compassion scale. Journal of health care for the poor and underserved. 2013;24(3):1235–46. 10.1353/hpu.2013.0148 23974394 PMC3915801

[32] Durkheim E, Fields KE. The elementary forms of religious life: Free Press New York; 1995.

[33] Thoits PA. Mechanisms linking social ties and support to physical and mental health. Journal of health and social behavior. 2011;52(2):145–61. 10.1177/0022146510395592 21673143

[34] Sprecher S, Fehr B. Enhancement of mood and self-esteem as a result of giving and receiving compassionate love. Current Research in Social Psychology. 2006;11(16):227–42.

[35] Banks R. Health and the spiritual dimension: relationships and implications for professional preparation programs. J Sch Health. 1980;50(4):195–202. 6900139 10.1111/j.1746-1561.1980.tb07373.x

[36] Brown I. Exploring the spiritual dimension of school health education. The Eta Sigma Gamman. 1978;10(1):12–6.

[37] Chapman LS. Developing a useful perspective on spiritual health: love, joy, peace and fulfillment. American journal of health promotion: AJHP. 1987;2(2):12–7. 10.4278/0890-1171-2.2.12 22208509

[38] Russell RD. A joust with Obie: Some comments on convictions held by Delbert Oberteuffer about health and health education. Health Education. 1984;15(2):3–7. 6444005

[39] Bellingham R, Cohen B, Jones T, Spaniol le R. Connectedness: some skills for spiritual health. American journal of health promotion: AJHP. 1989;4(1):18–31. 10.4278/0890-1171-4.1.18 22204354

[40] Elkins DN, Hedstrom LJ, Hughes LL, Leaf JA, Saunders C. Toward a humanistic-phenomenological spirituality definition, description, and measurement. Journal of humanistic Psychology. 1988;28(4):5–18.

[41] Koenig HG, George LK, Titus P. Religion, spirituality, and health in medically ill hospitalized older patients. Journal of the American Geriatrics Society. 2004;52(4):554–62. 10.1111/j.1532-5415.2004.52161.x 15066070

[42] Lawler-Row KA, Elliott J. The role of religious activity and spirituality in the health and well-being of older adults. Journal of health psychology. 2009;14(1):43–52. 10.1177/1359105308097944 19129336

[43] Cohen S, Janicki-Deverts D, Miller GE. Psychological stress and disease. JAMA: the journal of the American Medical Association. 2007;298(14):1685–7. 10.1001/jama.298.14.1685 17925521

[44] Powell LH, Shahabi L, Thoresen CE. Religion and spirituality. Linkages to physical health. The American psychologist. 2003;58(1):36–52. 12674817 10.1037/0003-066x.58.1.36

[45] Fraser GE, Sabate J, Beeson WL, Strahan TM. A possible protective effect of nut consumption on risk of coronary heart disease: the Adventist Health Study. Archives of Internal medicine. 1992;152(7):1416 1627021

[46] Ernecoff NC, Curlin FA, Buddadhumaruk P, White DB. Health Care Professionals' Responses to Religious or Spiritual Statements by Surrogate Decision Makers During Goals-of-Care Discussions. JAMA Intern Med. 2015;175(10):1662–9. 10.1001/jamainternmed.2015.4124 26322823

