# Supplementary figures and images for: Church attendance, allostatic load and mortality in middle aged adults
Source: PLoS One. 2017 May 16;12(5):e0177618. doi: 10.1371/journal.pone.0177618 (PMC5433740; doi:10.1371/journal.pone.0177618)

**S1 Fig. Supplemental Figure 1.**

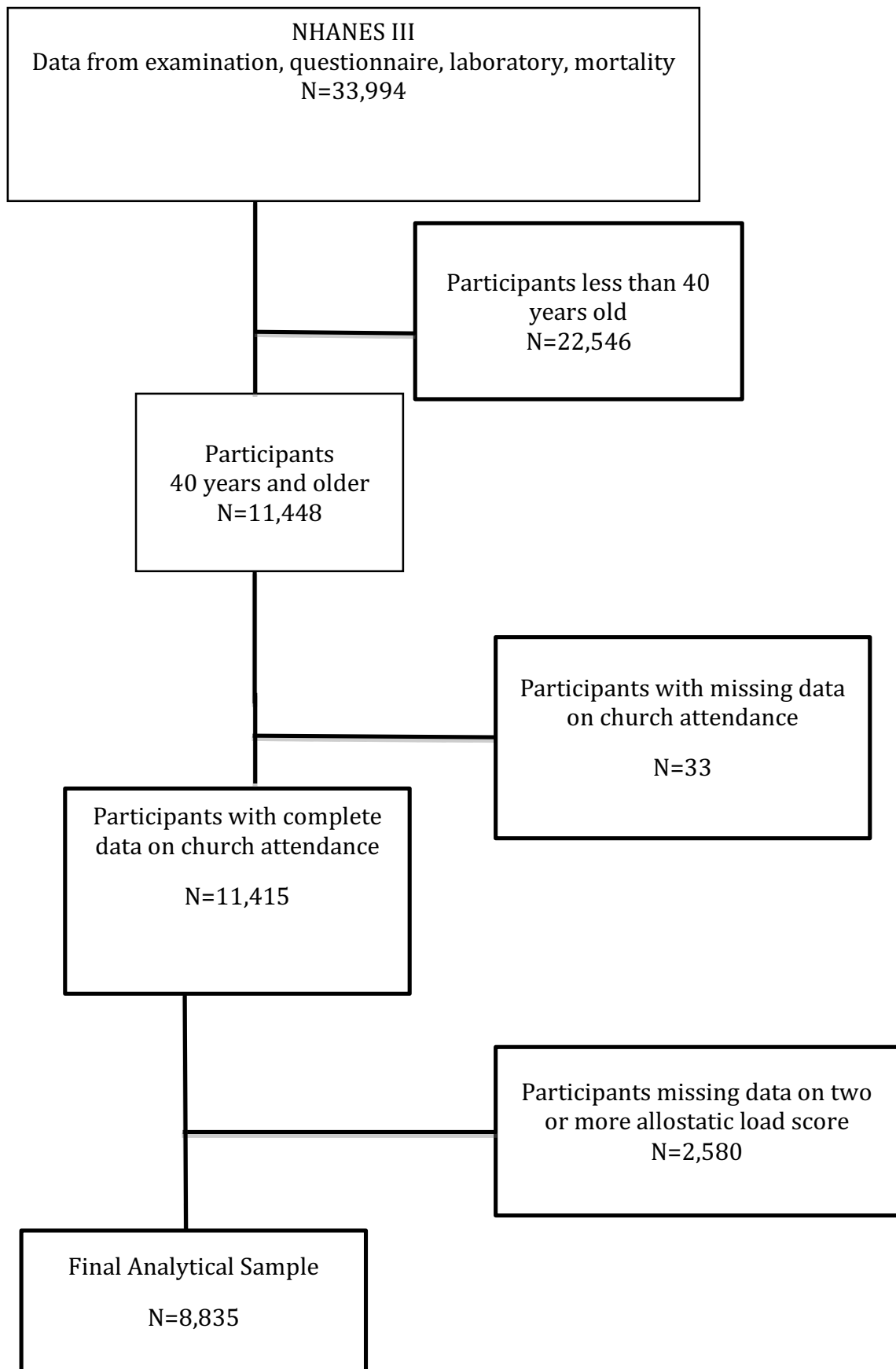

Supplement: S1 Fig — (PDF) [file pone.0177618.s001.pdf]

**S2 Fig. Supplemental Figure 2.**

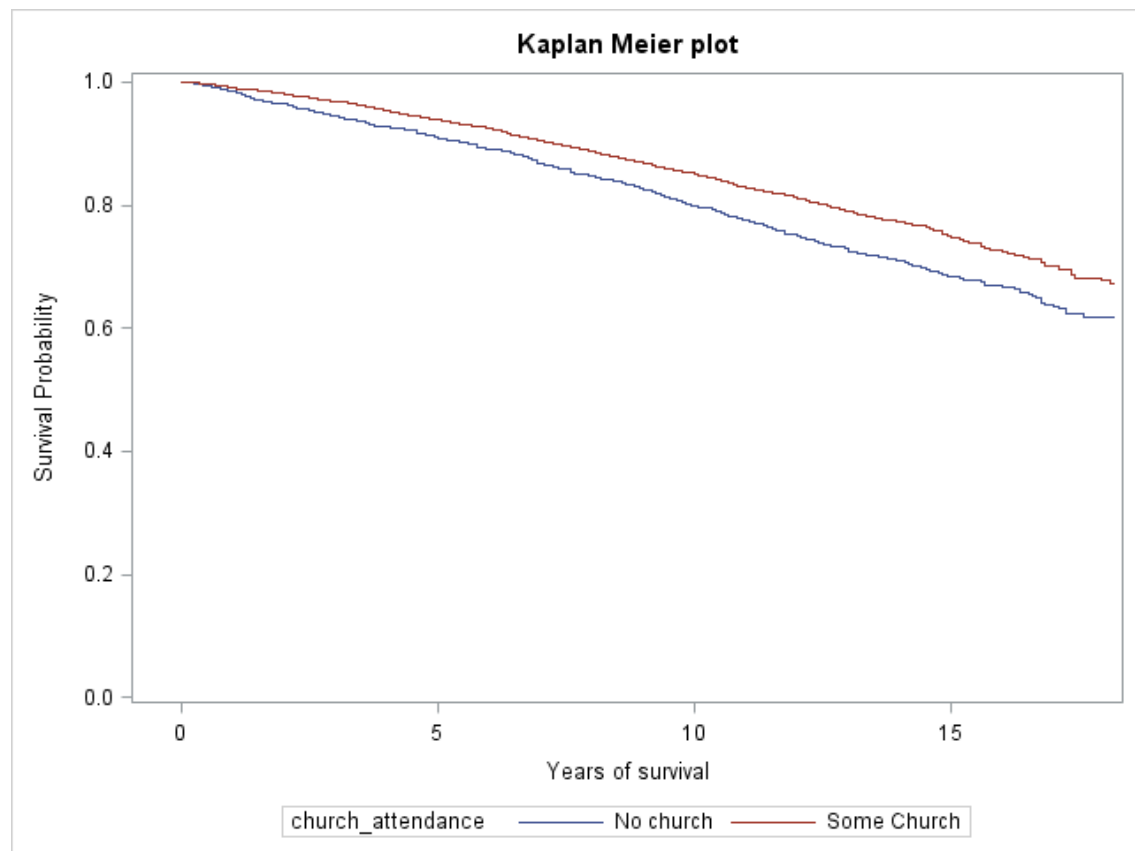

Supplement: S2 Fig — (PDF) [file pone.0177618.s002.pdf]
