# Supplementary material for: Church attendance, allostatic load and mortality in middle aged adults
Source: PLoS One. 2017 May 16;12(5):e0177618. doi: 10.1371/journal.pone.0177618 (PMC5433740; doi:10.1371/journal.pone.0177618)
Supplement: S1 Table — (DOCX) [file pone.0177618.s003.docx]

**S1 Table. Baseline characteristics of NHANES III population: demographics**

|  | **Total**  **(n=8835)** | **Churchgoers (At least once a year)**  **(*n*=6168)** | **Non- Churchgoers (< 1 time per year)**  **(*n*=2667)** | ***P* value** |
| --- | --- | --- | --- | --- |
| ***Demographics*** |  |  |  |  |
| Mean age (SE) years | 57(0.4) | 57(0.4) | 56(0.5) | 0.293 |
| **Race/Ethnicity**  White  Black  Hispanic  Other | 4475  2027  1997  336 | 2865(78.2)  1596(10.9)  1492(4.2)  215(6.7) | 1610(85.9)  431(5.6)  505(2.7)  121(5.8) | <0.0001 |
| **Sex**  Males  Females | 4270  4565 | 2718(43.6)  3450(56.4) | 1552(54.5)  1115(45.4) | <0.0001 |
| **Education**  <9 years (%)  9-12 years (%)  >12 years (%) | 2696  3361  2305 | 1873(15.3)  2318(41.6)  1658(43.1) | 823(17.0)  1043(46.9)  647(36.1) | 0.001 |
| Poor (poverty/income ratio<2) (%) | 3726 | 2600(27.1) | 1126(31.7) | 0.016 |
| No health insurance (%) | 853 | 571(6.1) | 282(10.2) | 0.002 |
| **Self-rated health**  Excellent/Very Good (%)  Good (%)  Fair/Poor (%) | 3066  3140  2623 | 2152(48.6)  2223(33.6)  1789(17.8) | 914(43.6)  917(33.6)  834(22.8) | 0.001 |
| **Social Support** Mean (SE) |  |  |  |  |
| In a typical week, how many times do you talk on the telephone with family, friends, or neighbors? (per week) | 8793 | 87(13.2) | 200.1(41.3) | 0.049 |
| How often do you get together with friends or relatives; I mean things like going out together or visiting in each other's homes? (per year) | 8831 | 110.8(3.5) | 105.3(7.2) | 0.0001 |
| About how often do you visit with any of your other neighbors, either in their homes or in your own? (per year) | 8828 | 65.1(3.4) | 66.5(7.5) | 0.009 |
| **Comorbidities (non-CV related)** |  |  |  |  |
| Lung disease (%) | 818 | 509(8.9) | 309(11.8) | 0.011 |
| Cancer (%) | 499 | 332(5.4) | 167(5.7) | 0.712 |
| Thyroid disease (%) | 631 | 464(8.1) | 167(7.1) | 0.312 |
| Rheumatoid arthritis (%) | 535 | 383(5.1) | 152(5.2) | 0.837 |
| Systemic lupus erythematosus (%)^a^ | 22 | 14(0.3) | 8(0.7) | 0.148 |
| Asthma (%) | 634 | 427(8.0) | 207(8.4) | 0.629 |
| **Health Behaviors** |  |  |  |  |
| Current smokers (%)  Former smokers (%)  Never smokers (%) | 1950  2943  3942 | 1136(18.6)  1970(34.3)  3062(47.1) | 814(31.4)  973(38.0)  880(30.6) | <0.0001 |
| Physically active (%) | 5876 | 4194(77.6) | 1682(73.3) | 0.002 |
| Non-drinkers (%)  1-30 alcoholic drinks/month (%)  >30 alcoholic drinks/month (%) | 5164  3073  592 | 3773(52.5)  2068(41.0)  324(6.*5) | 1391(46.9)  1005(41.3)  268(11.8) | 0.0001 |
| Mean (SE) Healthy Eating Index (HEI) score | 8524 | 66.1(0.4) | 63.9(0.5) | <0.001 |

^a^Estimate is unreliable, as the sample size was smaller than that recommended in the NHANES analytic guidelines for the design effect and estimated proportion.(22, 23)

The data presented are the weighted percentages, so they may not add up to 100.

SE: standard error; CV-cardiovascular
