# Supplementary material for: Church attendance, allostatic load and mortality in middle aged adults
Source: PLoS One. 2017 May 16;12(5):e0177618. doi: 10.1371/journal.pone.0177618 (PMC5433740; doi:10.1371/journal.pone.0177618)
Supplement: S2 Table — (DOCX) [file pone.0177618.s004.docx]

**S2 Table. Baseline characteristics of NHANES III population: allostatic load components**

|  | **Total**  **(n=8835)** | **Churchgoers (At least once a year)**  **(*n*=6168)** | **Non- Churchgoers (< 1 time per year)**  **(*n*=2667)** | *P* value |
| --- | --- | --- | --- | --- |
| ***Allostatic Load Components***  (% of each subgroup with “high risk” values)^a^ |  |  |  |  |
| Systolic blood pressure (SBP) | 2926 | 2015(23.2) | 911(26.1) | 0.075 |
| Diastolic blood pressure (DBP) | 716 | 502(6.8) | 214(8.4) | 0.054 |
| Waist/hip ratio (WHR) | 7172 | 4989(77.2) | 2183(80.1) | 0.106 |
| HDL | 2149 | 1421(23.3) | 728(28.1) | 0.001 |
| Total cholesterol/ HDL ratio | 3081 | 2089(33.3) | 992(38.0) | 0.006 |
| Glycated hemoglobin (HbA1c) | 3058 | 2176(24.2) | 882(24.8) | 0.589 |
| Heart Rate (HR) | 337 | 217(3.2) | 120(4.5) | 0.078 |
| Albumin (ALB) | 1303 | 942(13.3) | 361(10.9) | 0.055 |
| C-reactive protein (CRP) | 3484 | 2396(33.4) | 1088(35.1) | 0.382 |
| Body Mass Index (BMI) | 2412 | 1716(25.1) | 696(26.7) | 0.319 |
| Mean (SE) allostatic load score [range 0-10] | 8835 | 2.6(0.1) | 2.8(0.1) | 0.002 |

^a^High-risk values were defined as Systolic blood pressure>140 mmHg; Diastolic blood pressure>90 mmHg; waist/hip ratio>0.9(Males) waist/hip ratio>0.85(Females); HDL<40; Chol/HDL>5; HbA1c>5.7; Heart rate>90; albumin <3.8; C-reactive protein≥0.3; Body mass index >30; SE: standard error.
