# Supplementary material for: Church attendance, allostatic load and mortality in middle aged adults
Source: PLoS One. 2017 May 16;12(5):e0177618. doi: 10.1371/journal.pone.0177618 (PMC5433740; doi:10.1371/journal.pone.0177618)
Supplement: S3 Table — (DOCX) [file pone.0177618.s005.docx]

**S3 Table. Elevated allostatic load by church attendance**

| Allostatic load Score^a^ | Odds ratio (95% CI) of higher allostatic load score of No Church vs. Some Church |
| --- | --- |
| **2-3 vs. 0-1** | 1.14 (0.94-1.38) |
| **≥ 4 vs. 0-1** | 1.35 (1.11-1.63) ^b^ |

^a^Total allostatic load score was categorized using 3 different cutoffs (0-1, 2-3, and ≥4). Sum of the components include diastolic BP, systolic BP, HbA1c, waist to hip ratio, HDL, total cholesterol/HDL ratio, albumin, C-reactive protein, heart rate and BMI concentrations.

^b^*P*=0.003
