# Supplementary material for: Church attendance, allostatic load and mortality in middle aged adults
Source: PLoS One. 2017 May 16;12(5):e0177618. doi: 10.1371/journal.pone.0177618 (PMC5433740; doi:10.1371/journal.pone.0177618)
Supplement: S4 Table — (DOCX) [file pone.0177618.s006.docx]

**S4 Table. Hazard ratio for all-cause mortality by church attendance**

|  | **Unadjusted** | **Adjusted** | | | | |
| --- | --- | --- | --- | --- | --- | --- |
|  |  | Model 1 | Model 2 | Model 3 | Model 4 | Model 5 |
| **No church at all**  **(N = 2667)** | Reference | Reference | Reference | Reference | Reference | Reference |
| **Less than weekly**  **(N = 2085)** | 0.64  (0.53-0.76) | 0.74  (0.63-0.86) | 0.73  (0.62-0.87) | 0.79  (0.66-0.94) | 0.81  (0.68-0.95) | 0.77  (0.64-0.93) |
| **Weekly**  **(N = 3067)** | 0.91  (0.80-1.0) | 0.72  (0.64-0.80) | 0.71  (0.62-0.81) | 0.80  (0.68-0.93) | 0.83  (0.71-0.96) | 0.77  (0.60-0.98) |
| **More than weekly**  **(N = 1016)** | 0.71  (0.59-0.84) | 0.60  (0.50-0.72) | 0.62  (0.50-0.75) | 0.66  (0.53-0.83) | 0.71  (0.56-0.89) | 0.66  (0.49-0.87) |

Model 1 adjusts for age, sex, and race

Model 2 adds education, poverty-income ratio, and health insurance status to the covariates in Model 1

Model 3 adds health behaviors and the healthy eating index score to the covariates in Model 2

Model 4 adds allostatic load score to the covariates in Model 3.

Model 5 adds social support and self-rated health to the covariates in Model 4.

(All adjusted models adjusted for asthma, chronic obstructive pulmonary disease, non-skin cancer, thyroid disease, rheumatoid arthritis
